# Applications of multiphoton microscopy in imaging cerebral and retinal organoids

**DOI:** 10.3389/fnins.2024.1360482

**Published:** 2024-03-05

**Authors:** Macit Emre Lacin, Murat Yildirim

**Affiliations:** Department of Neurosciences, Cleveland Clinic Lerner Research Institute, Cleveland, OH, United States

**Keywords:** multiphoton/two-photon imaging, neurodevelopmental disorder, corticogenesis, cerebral organoid, multiphoton/three-photon imaging

## Abstract

Cerebral organoids, self-organizing structures with increased cellular diversity and longevity, have addressed shortcomings in mimicking human brain complexity and architecture. However, imaging intact organoids poses challenges due to size, cellular density, and light-scattering properties. Traditional one-photon microscopy faces limitations in resolution and contrast, especially for deep regions. Here, we first discuss the fundamentals of multiphoton microscopy (MPM) as a promising alternative, leveraging non-linear fluorophore excitation and longer wavelengths for improved imaging of live cerebral organoids. Then, we review recent applications of MPM in studying morphogenesis and differentiation, emphasizing its potential for overcoming limitations associated with other imaging techniques. Furthermore, our paper underscores the crucial role of cerebral organoids in providing insights into human-specific neurodevelopmental processes and neurological disorders, addressing the scarcity of human brain tissue for translational neuroscience. Ultimately, we envision using multimodal multiphoton microscopy for longitudinal imaging of intact cerebral organoids, propelling advancements in our understanding of neurodevelopment and related disorders.

## 1 Introduction

Research into neurodevelopment and neurological disorders gained significant momentum when it was discovered that human somatic cells could be reprogrammed into induced pluripotent stem cells (iPSCs), which then can be guided to differentiate into human brain cells (Takahashi and Yamanaka, 2006; Shi et al., 2012; Zhang et al., 2013). Initial attempts concentrated on differentiating hiPSCs into two-dimensional (2D) layers of adherent cells (Zhang et al., 2001; Chambers et al., 2009; Krencik and Zhang, 2011). However, researchers realized that 2D cultures had several drawbacks. First, the cultures generated this way lacked the cellular diversity in the human brain. Second, such preparations could not be kept alive for extended periods of time to study late neurodevelopmental processes like human corticogenesis. Third, these cultures were limited in mimicking the 3D architecture of the neural circuits (Levy and Paşca, 2023).

Recently, advances in differentiation and culturing techniques led to the development of stem cell-derived 3D models of the brain, which addressed several issues associated with 2D. Cerebral organoids are self-organizing multicellular structures generated by either unguided differentiation, resulting in a greater variety of cell types, or guided differentiation using patterning molecules to induce the formation of specific brain areas (Pașca et al., 2022). Unlike the 2D cultures, cerebral organoids can be maintained for months, allowing for the investigation of late neurodevelopmental processes such as migration, circuit-wiring, or long-range connectivity, which frequently go awry in disease conditions (Lancaster and Knoblich, 2014a; Pasca, 2018; Chen et al., 2020; Kelley and Pasca, 2022). Assembloids, on the other hand, are structures made by functionally integrating two or more organoids or an organoid with a different type of cell or tissue, such as muscle or immune cells (Birey et al., 2017; Pasca, 2019). Assembloids facilitate studying the interaction of different tissue types, such as the neuroimmune axis (Chen et al., 2020; Kelley and Pasca, 2022).

Despite these advantages, the lack of blood vessels in cerebral organoids proved a significant challenge for the researchers. The absence of a vasculature system to supply oxygen and nutrients and remove metabolites across the tissue results in a necrotic core in the cerebral organoid and limits its growth (LaMontagne et al., 2022; Ye, 2023). Moreover, such cerebral organoids have less differentiated cell types, less-developed cortical layers, and inconsistent electrical activity (Luo et al., 2016; Qian et al., 2019; Puppo and Muotri, 2023). Continuous agitation within the medium via spinning bioreactors can enhance nutrient diffusion into the organoid (Lancaster et al., 2013; Lancaster and Knoblich, 2014b; Li et al., 2023). However, this diffusion is usually limited, and constant agitation may hamper long-term live imaging of cortical organoids. Multiple strategies have been proposed to vascularize cerebral organoids. In *in vivo* approach, the cerebral organoid is transplanted into the brain of an immunodeficient rodent. The host vasculature then integrates into the grafted organoid (Mansour et al., 2018; Revah et al., 2022). *In vitro* strategies, on the other hand, involve either the co-culture of the cerebral organoid, gene editing, or fusion of vessel organoids with cerebral organoids. In the first of these approaches, human umbilical vein endothelial cells (HUVECs) are cultured with either human induced pluripotent stem cells (iPSCs) or embryonic stem cells before initiating neural differentiation. This process results in the formation of a vascular network by the HUVECs within the cerebral organoids (Shi et al., 2020). An alternative to this approach involves embryoid bodies comprising both unmodified iPSCs and iPSCs equipped with doxycycline-inducible ETS variant transcription factor 2 (ETV2). ETV2 encodes for a protein that initiates tubulogenesis pathways, and its induction during cerebral organoid differentiation results in the formation of an extensive, branched vascular network throughout the organoid (Cakir et al., 2019). Finally, another strategy involves fusing cerebral and vessel organoids. This yields a vascularized cerebral organoid that displays blood–brain-barrier-like structures and contains immune cells such as microglia (Sun et al., 2022).

Cerebral organoids are often studied by microscopic imaging and analysis. Most imaging studies with cerebral organoids involve a fixation step. The fixed cerebral organoid is then commonly sliced to facilitate imaging. Imaging in fixed organoids often involves immunolabeling a cellular antigen with a fluorescent tag. Immunolabeling can identify various structural components such as microtubules or nuclei or different cell types such as astrocytes, oligodendrocytes, or dopaminergic cells (Martin et al., 2021). Fixed cerebral organoids can also be imaged intact form without slicing. For this, various clearing protocols have been developed (Matryba et al., 2019). Other imaging studies focus on dynamic events in live cerebral organoids, such as Ca^+2^ activity or neuronal migration (Yildirim et al., 2022). In this paper, we will concentrate on studies that involve live organoid imaging.

Multiple imaging approaches have been employed to study the neurodevelopmental processes in the cerebral organoids. However, several challenges and limitations are impacting intact organoid imaging. First, the millimeter size of mature organoids and the compact organization of organoid cell layers impede light penetration (Keshara et al., 2022). Second, organoids comprise various biomolecules such as proteins, lipids, water, and minerals with wide-ranging refractive indices, resulting in substantial light scattering and absorption. Owing to these limitations, most cerebral organoid imaging studies rely on imaging sliced, very thin sections or superficial parts of cerebral organoids (Tuchin et al., 1997; Yu et al., 2011; Feuchtinger et al., 2016; Grist et al., 2016; Seo et al., 2016; Kumar et al., 2023).

Limited access to human brain tissue for studying human-specific phenomena and brain disorders is a significant barrier in translational neuroscience. Human cerebral organoids and assembloids are beginning to address this deficit by giving access to neural preparations that can be studied in detail. Multiple imaging methods have been employed to visualize the morphogenesis and differentiation of organoids. Currently, most common methods rely on one-photon excitation. However, opacity, millimeter-scale sizes, and tight organization of cell layers of organoids impose strict limits on the image resolution and contrast, especially for deeper regions of intact cerebral organoids. Multiphoton microscopy (MPM) relies on non-linear excitation of fluorophores and employs longer wavelengths than one-photon imaging methods. These characteristics make MPM a good fit for imaging highly scattering live cerebral organoids. Here, we first describe the standard microscopy techniques for cerebral organoid imaging and point to the limitations of each. We then introduce multiphoton microscopy (MPM) as an alternative to address some of the challenges associated with imaging highly scattering, thick, and intact cerebral organoids. Next, we review recent applications of MPM in imaging cerebral organoids. Finally, we discuss future possibilities for using multimodal multiphoton microscopy to image intact cerebral organoids longitudinally.

## 2 Microscopy techniques for imaging cerebral organoids

**Figure 1:**
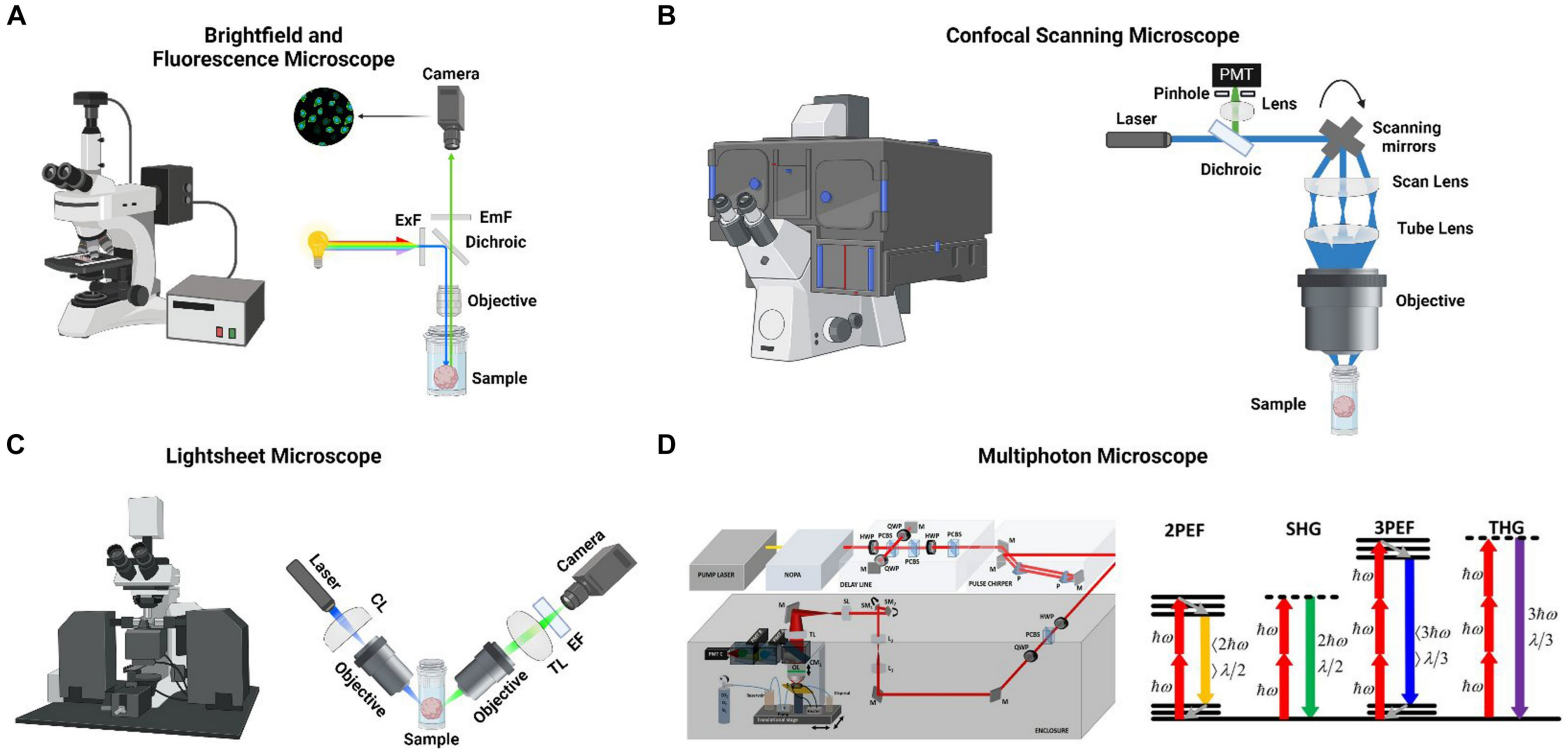
**(A)** (Left) A commercial brightfield and fluorescent microscope. (Right) Schematic of a fluorescent microscope where large spectral bandwith light source is filtered for a specific wavelength and excites a cerebral organoid with a large field of view and then emitted light is collected with a camera. **(B)** Xue et al. monitored the metabolic, structural and molecular changes in differentiating retinal organoids with Fluorescence life-time imaging microscopy (FLIM) and hyperspectral imaging (Xue et al., 2021). In the mature retinal organoid (D169) the glycolytic surface is limited to the layer where photoreceptor cells are located (left). The retinol concentrates in the same region over the course of development (left). Long-term FLIM tracking of free to bound NADH ratio revealed that a transition from glycolysis to oxidative phosphorylation occurs around the 2nd and 3rd month of culture (right). **(C)** (Left) A commercial light sheet microscope. (Right) Schematic of a light sheet microscope where visible light’s axial resolution is elongated with a cylindrical lens and focused to a cerebral organoid. Emitted light is collected with another objective lens and focused to a camera with a tube lens. **(D)** (Left) Schematic of an custom-made three-photon microscope for performing live cerebral organoid imaging. Femtosecond laser pulses from a pump laser (1,045 nm) were pumped through a noncollinear optical parametric amplifier (NOPA) to obtain 1,300 nm excitation wavelength. Laser beams were scanned by a pair of galvanometric scanning mirrors (SM), and passed through a scan lens (SL) and a tube lens (TL) on the back aperture of a 1.05 NA, 25 × objective. Emitted light was collected by a dichroic mirror (CM1), collection optics (CO), laser blocking filters (BF), and nonlinear imaging filters (F) and corresponding collection optics (COA, COB, and COC) for each photomultiplier tube (PMT A, PMT B, and PMT C) (Adapted from Yildirim et al., 2022). (Right) Energy-level diagrams of different kinds of multiphoton microscopes (MPM), such as 2PEF, SHG, 3PEF, and THG. ℎ𝜔 is the excitation photon energy, 𝜆 denotes wavelength (Adapted from Srinivasan and Yildirim, 2023). Panels **(A-C)** are designed by @Biorender.

Bright-field, fluorescence, confocal, and light-sheet microscopy are the most common microscopy techniques for imaging cerebral organoids (Martin et al., 2021) (Figure 1). Each method has unique advantages and limitations.

Bright-field (BF) microscopy, which depends on light transmitted through the sample, is in general used to evaluate 3D brain culture shape and surface characteristics (Iefremova et al., 2017; Monzel et al., 2017) (Figure 1A). However, as cerebral organoids are often too thick to allow light penetration, BF microscopy is not well suited for observing individual cells within the organoid.

Confocal microscopy (CM) is the most common fluorescence microscopy method for 3D brain cultures (Martin et al., 2021) (Figure 1B). Most CM techniques rely on laser illumination of the specimen and pinhole filtering of the emitted light to remove out-of-focus light. CM allows imaging at sub-micrometer resolution, and optical sectioning of thicker specimens. These enable visualization of organoid features, such as surface characteristics, 3D shape, and cellular distribution (Qian et al., 2016; Raja et al., 2016; Monzel et al., 2017; Karzbrun et al., 2018). However, due to light scattering, the penetration depth of single-photon techniques such as confocal microscopy is typically limited to <100 μm. The resolution of confocal microscopy is only adequate for the most superficial layers of the organoid and significantly deteriorates in deeper layers (Smits and Schwamborn, 2020). Laser-induced phototoxicity and photobleaching can impede long-term imaging (Peel and Jackman, 2021). Finally, CM recording sessions require a considerable amount of time, up to several hours per organoid, for in-depth imaging. Confocal microscopy typically identifies proteins or molecules labeled with fluorescent markers, such as antibodies or stains. Immunolabeling requirements impose several limitations on confocal microscopy. Achieving homogenous labeling of cells within cerebral organoids presents a significant challenge, owing to cerebral organoids’ dense, three-dimensional architecture and inherent heterogeneity in size, structural complexity, and cellular composition (Keshara et al., 2022; Zhao et al., 2023). Secondly, imaging deep within cerebral organoids is limited due to light scattering and absorption (Keshara et al., 2022). Third, fluorescent labels can suffer from photobleaching during prolonged imaging, and the light imaging can be phototoxic to cells, affecting their viability and behavior (Fei et al., 2022). Owing to the difficulty in obtaining selective and well-validated antibodies, endogenous protein detection is typically restricted to a limited number of proteins and necessitates fixed tissue imaging. Maintaining the viability of cerebral organoids during live imaging over extended periods is challenging due to the need to control for temperature, CO_2_ levels, and humidity (Keshara et al., 2022). Moreover, live imaging frequently requires the attachment of large fluorophores to proteins, which can disrupt their function (Specht et al., 2017). Specifically designed fluorescent protein (FP)-based constructs address some of these issues. They cause minimal disruption to cellular mechanisms and enable visualization of dynamic events in live tissue (Dong et al., 2022). For instance, genetically encoded calcium indicators (GECIs) such as GCaMP and its red variant RGECO enable monitoring intracellular calcium levels as a surrogate for electrical activity (Tian et al., 2009; Chen et al., 2013; Dana et al., 2016). Similarly, genetically encoded voltage indicators (GEVIs) report neuronal spiking and sub-threshold voltage changes (Knöpfel and Song, 2019). Neurotransmitter and neuromodulator sensors such as iGluSnFR and GRAB_DA_ allow imaging neurotransmitter release with high molecular specificity and spatiotemporal resolution (Helassa et al., 2018; Patriarchi et al., 2018; Sun et al., 2018). On the other hand, other FP-based techniques, such as cell-type specific promoter-driven FP expression, can facilitate identifying cell types and enable tracking of neuronal migration. Similarly, recently developed Brainbow and Tetbow techniques, which rely on the stochastic expression of multiple FPs, permit analyses of neuronal connectivity, cell migration, and lineage (Livet et al., 2007; Cai et al., 2013; Sakaguchi et al., 2018).

Light sheet (LS)- fluorescence microscopy has several advantages over point-scanning confocal microscopy, including faster acquisition, reduced scattered light, and less photobleaching and phototoxicity to cells (Huisken et al., 2004; Delgado-Rodriguez et al., 2022) (Figure 1C). In LS microscopy, a sheet of light is produced within the sample that aligns with the focal plane of a high-NA objective orthogonal to the light sheet (Peel and Jackman, 2021). Based on the original single-sided light-sheet illumination, many varieties of LS imaging have been created, including lattice and dual-illumination light sheets (Huisken et al., 2004; Chen et al., 2014; de Medeiros et al., 2015). Despite the advantages of LS over confocal microscopy, only a small number of cerebral organoid studies employ this imaging method for the following reasons: (1) LS microscopy requires a long, complex clearing protocol before the imaging session and, in general, testing different clearing techniques is necessary before obtaining high-quality images (Martin et al., 2021), (2) moreover, light penetration and resolution in deeper regions are insufficient for reconstructing a 3D organoid connection map (Poli et al., 2019), (3) LS microscopy mostly requires the fixation of cerebral organoids, and it is limited to image of superficial layers of live cerebral organoids (He et al., 2022).

Compared to the other microscopy techniques, two-photon (2P) microscopy significantly enhances the three-dimensional contrast and resolution, particularly when imaging deep in environments with high scattering, such as cerebral organoids. 2P microscopy achieves this by (1) utilizing longer excitation wavelengths to reduce scattering and (2) establishing nonlinear (two-photon) processes between light and tissues to reduce out-of-focus light generation. First, the excitation wavelengths used in 2PE microscopy, deep red and near-infrared, penetrate tissue more effectively than visible wavelengths owing to reduced scattering and absorption by endogenous chromophores (Svoboda and Block, 1994; Oheim et al., 2001; Yaroslavsky et al., 2002). Second, 2P excitation of a fluorescent molecule is a non-linear process, so the emitted fluorescence signal increases fourfold with a doubling of laser intensity. And the fluorescence is greatest close to the focal point and decreases quartically (fourth power) with axial distance. Consequently, fluorophores are excited almost exclusively in a small focal volume of 0.1–0.3 um^3^ based on numerical aperture range of 1–1.2 with water immersion lens at the wavelength of 900 nm (Zipfel et al., 2003). Also, scattered photons are too dilute to excite out-of-focus fluorophores and create a background noise. Lastly, due to excitation localization, all fluorescence photons, including the scattered, constitute useful signals if detected (Svoboda and Yasuda, 2006).

Three-photon microscopy is another non-linear optical microscopy technique that has gained prominence in neuroscience research in recent years (Yildirim et al., 2019, 2020, 2022) (Figure 1D). 3P excitation has several advantages over 2P, allowing for deeper imaging in scattering tissue. First, 3 PM uses longer excitation wavelengths than 2P, which attenuates less in the tissue (Wang et al., 2018; Xiao et al., 2023). Second, 3 PM displays higher order non-linearity than 2P, reducing the out-of-focus signal (Wang et al., 2020). Three-photon fluorescence microscopy has been recently used to perform structural and functional brain imaging in anesthetized and awake mice (Ouzounov et al., 2017; Yildirim et al., 2019). These studies utilized a green (GCaMP) genetically engineered calcium indicator (exogenous fluorophores) with their excitation wavelength (1,300 nm), which provides peak absorption cross-sections for this indicator.

Most 2P and 3P excitation studies rely on fluorescent dyes and proteins however imaging auto-fluorescent molecules such as flavin adenine dinucleotide (FAD) and nicotinamide adenine dinucleotide (NADH) addresses several issues associated with fluorescent probes. First, label-free imaging does not require the diffusion of a dye to the tissue and, therefore, is immune to uneven labeling of the tissue. Second, auto-fluorescent molecules are less prone to photobleaching, which is often a problem for long recordings of fluorescent probes. Third, the intracellular events are less affected, as label-free imaging does not necessitate transgene expression (Keshara et al., 2022). Label-free imaging of autofluorescent metabolic molecules can inform about the metabolic state of the organoid, which is important for evaluating organoid maturation (Zhao et al., 2023) (Figure 1D).

The two other label-free non-linear optical imaging modalities used in the 3D culture studies include second harmonic generation (SHG) and third harmonic generation (THG) (Zhao et al., 2023). These techniques deposit no energy on the tissue sample and, thus, reduce the probability of phototoxicity (Yelin and Silberberg, 2000; Campagnola and Loew, 2003). SHG necessitates intense femtosecond laser pulses passing through a highly polarizable material with a noncentrosymmetric molecular organization like collagen fibers (Campagnola and Loew, 2003). Conversely, THG can be applied to all molecules wherein three photons at the fundamental frequency are converted to a photon at the third harmonic frequency. The signal intensity in THG is very sensitive to the index of refraction and third-order susceptibility (χ^3^) changes (Barad et al., 1997; Muller et al., 1998). As a third-order nonlinear order (NLO) process, THG involves the conversion of three photons into a single photon with three-times the energy of the excitation photons. THG signal arises at the interface between water and lipids, such as cell membranes and lipid droplets, where there is a significant refractive index change (Zhao et al., 2023) (Figure 1D).

## 3 Disease modeling

Cerebral organoids and assembloids have been frequently used to study neurological diseases *in vitro*. Multiphoton microscopy facilitates the detection of disease symptoms such as cell migration deficits, network activity abnormalities, and cell differentiation deficits (Chen et al., 2020; Kelley and Pasca, 2022; Levy and Paşca, 2023). Several studies used multiphoton microscopy to show the validity of their organoid generation protocol, i.e., they can generate neurons with mature morphology with extensive neurites and capable of firing. For instance, Watanabe et al. created a new organoid development method (Watanabe et al., 2017). They employed 2P calcium imaging to show that they can generate physiologically active neurons. Similarly, Brady et al. developed basal ganglia organoids to study neurodevelopmental defects in Tourette’s syndrome. To demonstrate that the cortical organoid neurons were mature at the 3-month mark, they studied the structure of GFP-expressing neurons with 2 PM. They observed that the neurons had extensive branching and well-developed spines. Linkous et al. used cerebral cortical organoids and patient-derived glioma stem cells to model glioblastoma *in vitro* (Linkous et al., 2019). Labeling glioma stem cells with GFP and employing 2 PM to image deep into the cerebral organoid demonstrated that glioma cells can infiltrate the cerebral organoid.

**Figure 2:**
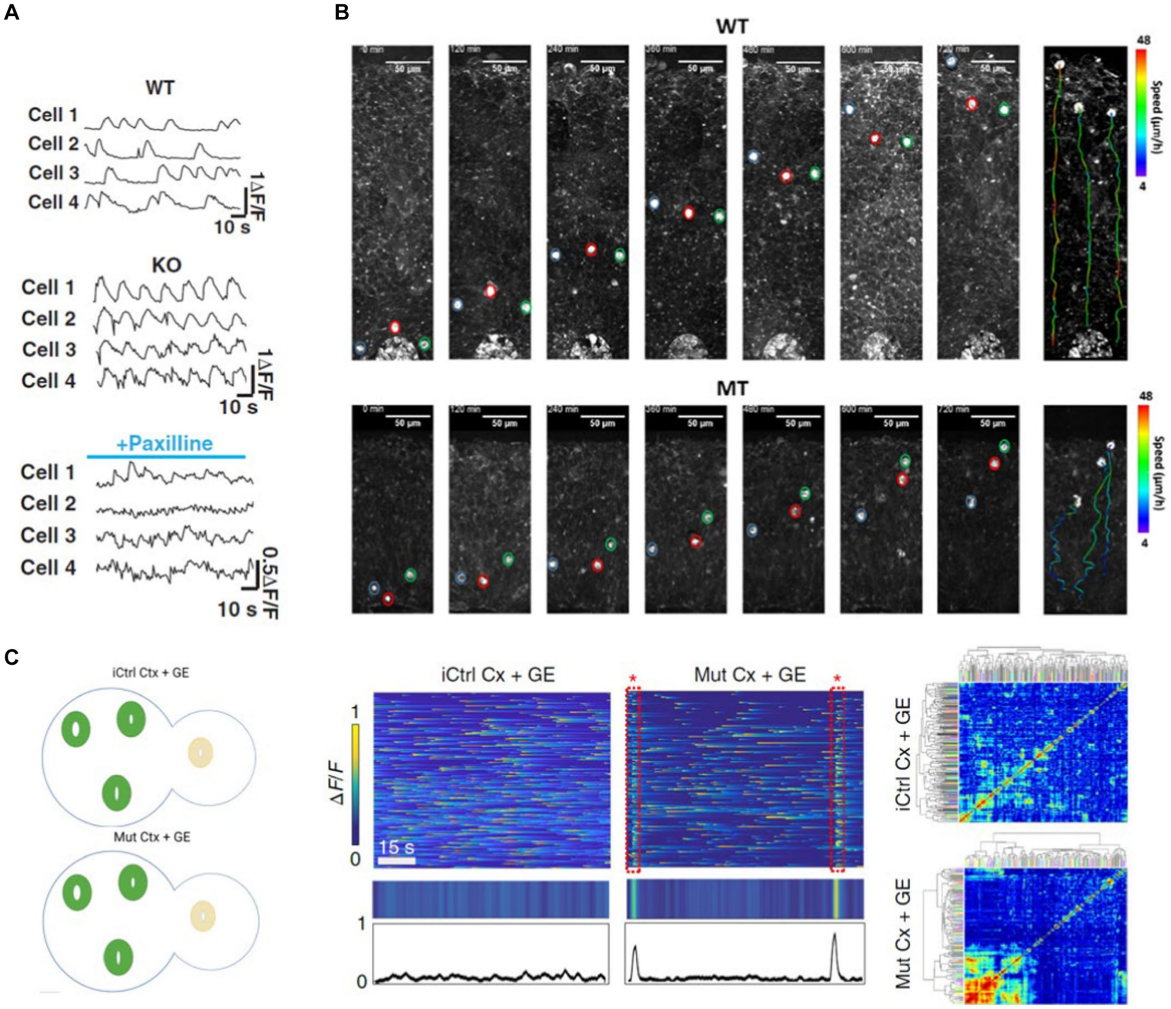
**(A)** Cortical Organoids (CO) derived from Angelman Syndrome patients show neuronal network synchrony (KO) (middle) which is absent in cortical organoids derived from healthy subjects (WT, top). The disease state network synchrony can be rescued by Paxilline (Sun et al., 2019) (bottom). **(B)** 3P label-free imaging of radially migrating neurons in COs derived from Rett Syndrome patients (MT, bottom) follow a more tortuous path with a slower speed compared to organoids derived from healthy subjects (WT, top) (Yildirim et al., 2022). **(C)** Assembloids generated by fusing cortical (CO) and ganglionic eminence (GE) organoids have both excitatory and inhibitory neurons and better recapitulate network abnormalities (left). Samaringhe et al. showed *MECP2*-mutant CO + GE assembloids derived from Rett patients exhibited spontaneously synchronized calcium transients like the epileptiform events observed in these patients (middle and right) (Samarasinghe et al., 2021).

Angelman syndrome is a disease characterized by seizures. Cortical organoids generated by knocking out a particular ubiquitin ligase associated with Angelman syndrome showed neuronal network hypersynchrony like human patients (Sun et al., 2019). Incubating Angelman cortical organoids in a potassium channel blocker, Sun et al. demonstrated that the frequency of Calcium activity and synchronous firing of neurons could be brought to wild type levels (Figure 2A) (Sun et al., 2019). To study the network activity of cortical organoid neurons, Sun et al. labeled the neurons with a synthetic Calcium dye. They took a time-lapse recording of the neuronal activity (Sun et al., 2019). Multiphoton imaging also enables researchers to study neurodevelopmental defects in unprecedented detail. Employing label-free 3P microscopy in cortical organoids derived from Rett syndrome patients, Yildirim et al. demonstrated that the ventricular zone area was larger, and its thickness was smaller in Rett organoids compared to control organoids (Yildirim et al., 2022). Moreover, using an integrated incubator chamber in their microscope, they performed long-term imaging of radially migrating neurons from the ventricular zones. This study demonstrated that these Rett organoid neurons follow a more tortuous path with a slower speed than healthy controls, resulting in shorter migration distances (Yildirim et al., 2022) (Figure 2B).

Cortical organoids lack diverse neuron types. Brain disorders associated with network abnormalities are better recapitulated by organoids both excitatory and inhibitory neurons. Samarisinghe et al. generated cortical (CO) and ganglionic eminence (GE) organoids and fused them to develop an “assembloid” in which excitatory and inhibitory neurons integrate (Samarasinghe et al., 2021). Their study showed that *MECP2*-mutant CO + GE assembloids derived from Rett patients exhibited spontaneously synchronized calcium transients similar to the epileptiform events observed in these patients (Figure 2C) (Samarasinghe et al., 2021). Moreover, the average amplitude of synchronized transients and the proportion of serially spiking neurons were increased in the mutant assembloids. Similarly, Kodera et al. generated cortical and ganglionic eminence organoids from marmoset ES cells and fused them to form an assembloid (Kodera et al., 2023). Like the developing embryo, they observed that inhibitory neurons migrate from the ganglionic eminence to the cortical organoid. Strikingly, two-photon calcium imaging in cortical organoids expressing jGCaMP demonstrated that synchronous neural firing observed during the immature state is replaced by nonsynchronous activity as the assembloid matures. Thus, cortical organoids can mimic developmental changes in spontaneous neural activity patterns.

Assembloid methodology is also applied to study oligodendrocyte formation in the developing brain *in vitro*. Kim et al. generated ventral and dorsal forebrain organoids using OLIG2-GFP knockin human pluripotent stem cell (hPSC) reporter lines and fused them to form forebrain assembloids (Kim et al., 2019). To demonstrate that they obtained physiologically active neurons and glial cells, they performed two-photon calcium imaging on the organoids. At week 6, they observed both cell types, which they could differentiate by the duration, number, and amplitude of the transients.

## 4 Transplantation

The existing procedures to grow cerebral organoids continue to encounter several difficulties, such as insufficient maturation and cellular stress. The absence of a microvasculature system in the organoid leads to decreased oxygen levels and limited nutrition delivery to the central region of the organoid. Therefore, the core of the organoid becomes susceptible to necrosis (Mansour et al., 2018; Qian et al., 2020). To overcome this issue, researchers began transplanting cerebral organoids into highly vascularized regions of animal hosts. Transplantation offers several advantages. First, physiological perfusion of the organoid by the host’s vessels prevents necrosis in the core and allows long-term organoid culture (Wang et al., 2023). Moreover, it provides organoids with nutrients that are often missed in *in-vitro* cultures.

**Figure 3:**
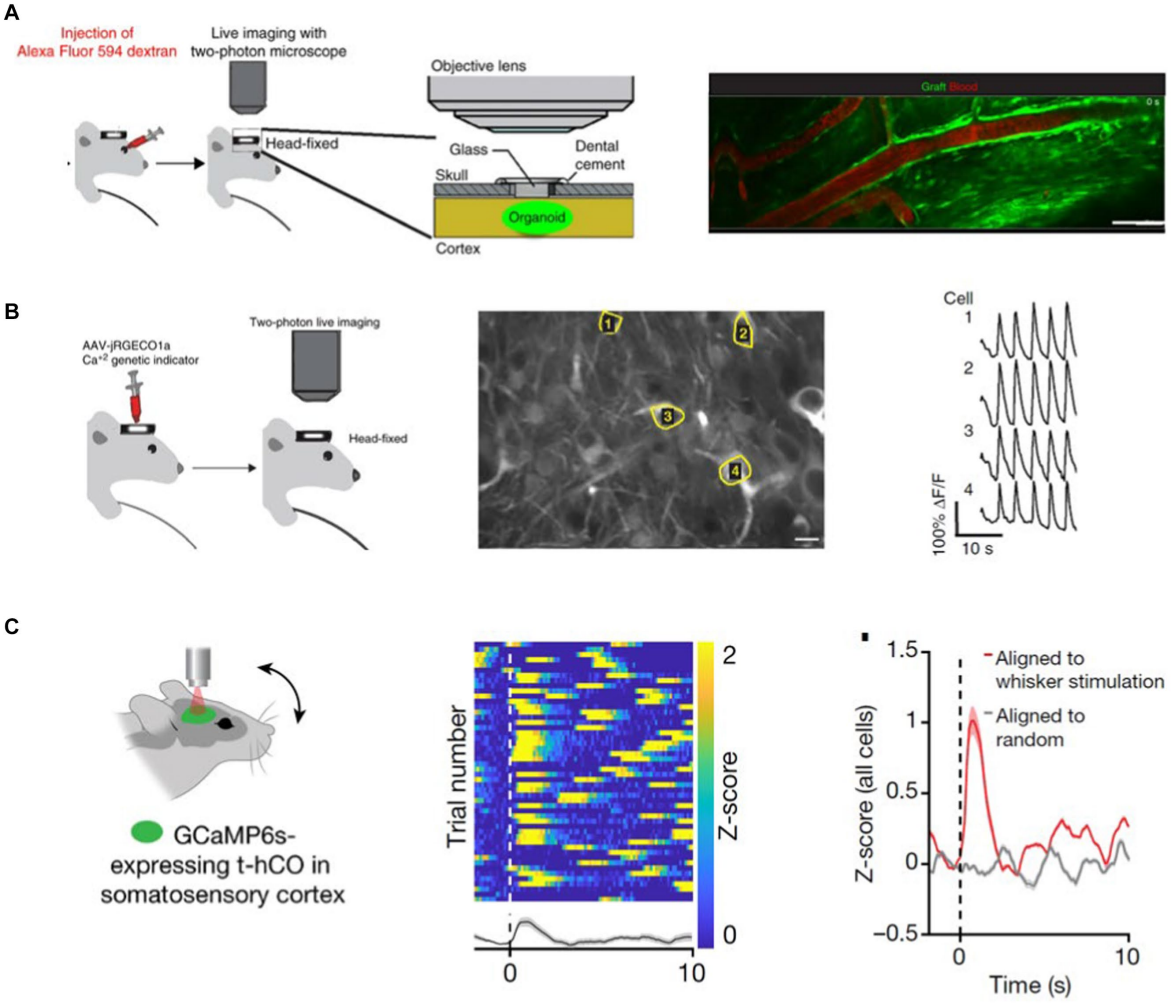
**(A)** GFP-expressing transplanted CO is vascularized by the host organism vessels. To demonstrate this, Mansour et al. RO-injected a Alexa 594 dye and observed the dye flow through the GFP-labeled CO with 2 PM (Mansour et al., 2018). **(B)** Expressing a red Calcium sensor in the GFP-labeled transplant Mansour et al. show that CO neurons are spontaneously active (Mansour et al., 2018). **(C)** Transplanted human COs integrates into the host rat’s S1 cortex and responds to somatosensory stimuli (Revah et al., 2022).

In an early study, Mansour et al. placed GFP-expressing organoids into a cavity made in the retrosplenial cortex of an immunodeficient mouse (Mansour et al., 2018). Mansour et al. demonstrated that host vessels vascularized the organoids by retro-orbitally injecting Alexa Fluor 594 dextran and imaging the red dye infiltration into the green fluorescent cortical organoid (Figure 3A). Moreover, they inject AAV.CaMKII.jRGECO1 viral vector into the grafted CO and 2P imaging of calcium activity, Mansour et al. showed that CO neurons are functional (Figure 3B). Taking a step further, Revah et al. evidenced that transplanted human cortical organoids can respond to somatosensory stimuli (Revah et al., 2022). For this, Revah et al. implanted human cortical organoids expressing GCaMP6s into the rat Somatosensory cortex (S1). Deflection of the whiskers contralateral to the S1 organoid implantation site activated a subset of the human CO neurons. These changes in activity were not observed when calcium activity data were aligned to randomized timestamps. These results indicate that transplanted CO integrates into the host’s brain network and can be activated by environmental stimuli (Figure 3C).

Vascularization is required for the organoid’s oxygen, nutrient, and waste exchange. Cerebral organoids have a size limit, beyond which necrosis becomes visible in the core of the cerebral organoid (Lancaster et al., 2013). To address this issue, Shi et al. generated vascularized organoids (vOrganoids), where they co-cultured the hES cells or hIPSCs with human umbilical vein endothelial cells (HUVECs) *in vitro* (Shi et al., 2020). They demonstrated that HUVECs form an intricate vascular system in the cerebral organoids. Moreover, when the vOrganoid is transplanted into the mouse S1 cortex, vOrganoid vessels are integrated into the host’s vascularization system. Live imaging with a 2P microscope following tail vein injection of Alexa Fluor 594 dextran showed that the dye ran through the organoid, demonstrating the host-graft vasculature integration. Similarly, Wilson et al. (2022) employed two-photon imaging of intravascular tracer Alexa 680-Dextran to demonstrate the host’s vascularization of the human CO graft (Wilson et al., 2022). To test the functional integration of the grafted human cortical organoid with the host mouse cortex, Wilson et al. recorded the visual stimuli-evoked neural activity in the grafted organoid with transparent microelectrode arrays (Wilson et al., 2022). Their studies showed that organoid neurons respond to sensory stimuli, and the surrounding host tissue modulates their activity.

## 5 Retina

**Figure 4:**
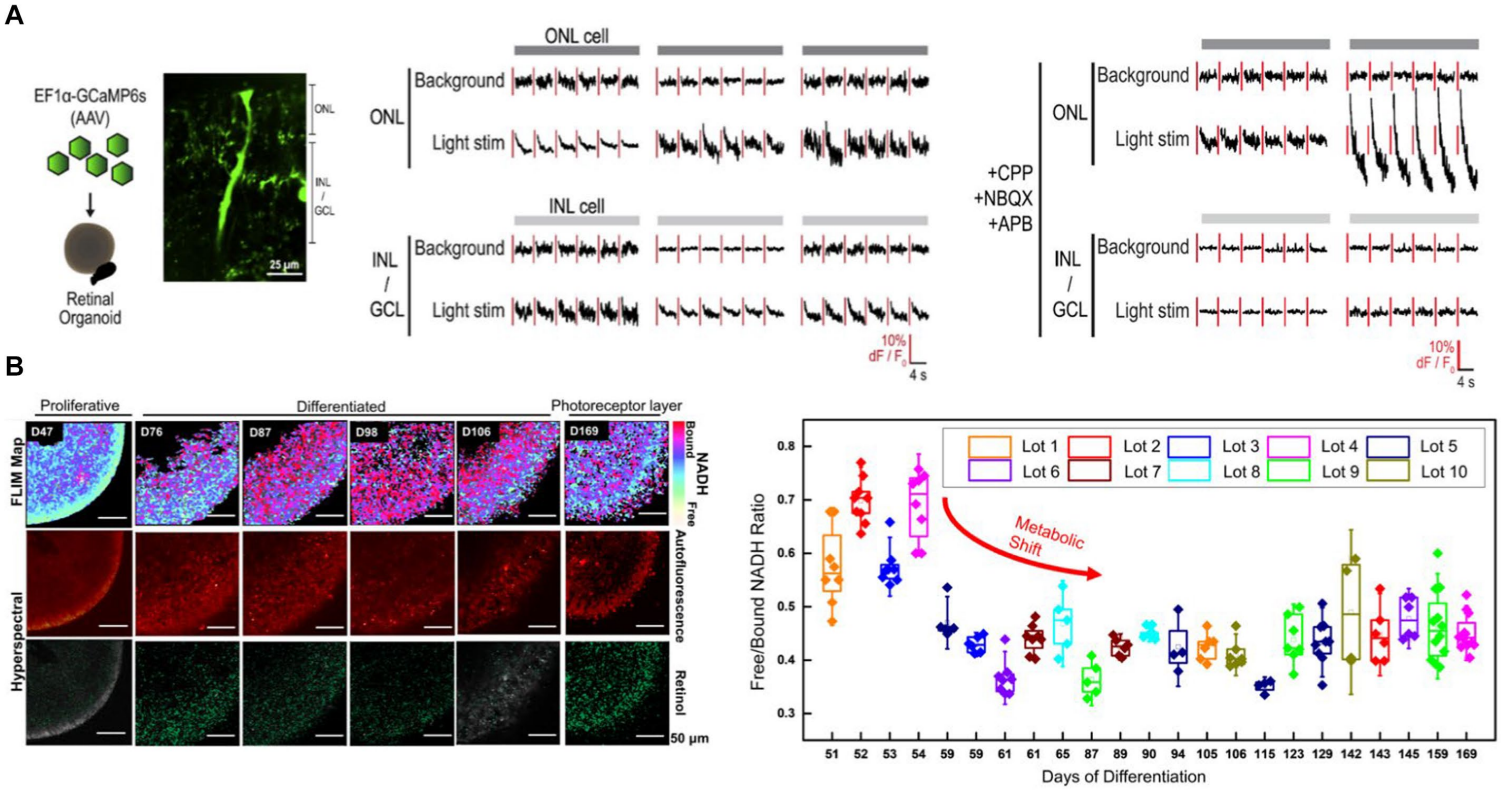
**(A)** Cowan et al. transduced retinal organoids with GCaMP6s and imaged across all three retina layers with 2P microscopy (left). Light stimulation evoked responses in the outer nuclear layer (ONL) as well as inner layer cell (INL and GCL) cells (Cowan et al., 2020) (middle). Light-evoked inner layer cell responses are abolished with glutamatergic antagonists confirming the necessity of glutamatergic transmission from the photoreceptors in the ONL to the inner retinal layers (right). **(B)** Xue et al. monitored the metabolic, structural and molecular changes in differentiating retinal organoids with Fluorescence life-time imaging microscopy (FLIM) and hyperspectral imaging (Xue et al., 2021) (left). Long-term FLIM tracking of free to bound NADH ratio revealed that a transition from glycolysis to oxidative phosphorylation occurs around the 2nd and 3rd month of culture (right). In the mature retinal organoid (D169) the glycolytic surface is limited to the layer where photoreceptor cells are located. Hyperspectral imaging of retinol overlaps with the photoreceptor layer.

Cowan et al. created a high-throughput method to develop retinal organoids from hiPSCs (Cowan et al., 2020). The organoids have all three retinal layers and transmit light responses to the photoreceptors in the outer layer to second and third-order retinal cells. Cowan et al. transduced the retinal organoid with AAV expressing GCaMP6s and performed 2P laser imaging across all three retinal layers to demonstrate this light transmission (Figure 4A, left panel) (Cowan et al., 2020). Light stimulus hyperpolarized 17% of the photoreceptors located in the outer nuclear layer. Moreover, light stimulus is transmitted to the inner layers of the retina, with 12% of the inner nuclear and ganglion layer cells consistently responding to light above background levels (Figure 4A, mid-panel). These responses were blocked by glutamatergic antagonists, indicating that the light-evoked responses in inner layer cells are driven by glutamatergic synaptic connections from the outer layer photoreceptors (Figure 4A, right panel).

Non-invasively assessing the retinal organoid’s developmental state is essential for the reproducibility of organoid-based pre-clinical and clinical studies. Xue et al. performed label-free 2P-based techniques to non-invasively monitor the retinal organoid’s metabolic, structural, and molecular changes during its development (Xue et al., 2021). Fluorescence lifetime imaging microscopy (FLIM) enabled Xue et al. to image free (f) and bound (b) NADH in the organoid. It demonstrated that a metabolic shift happens around 2 and 3 months of culture, during which the retinal organoid switches from predominantly glycolytic to an oxidative state, evidenced by a reduction in f/b NADH ratio (Figure 4B) (Xue et al., 2021). This shift is consistent with a decrease in proliferative activities and maturation of the retinal organoid. At the mature state of the retinal organoid (day169), the glycolytic surface is limited to where photoreceptor cells are located. Hyperspectral imaging of retinol, which is a marker of photoreceptor cells, confirms the FLIM findings. The retinol signal starts as a diffuse signal in the inner layer. The retinol gradually condenses around the outer layers as the organoid differentiates, creating a distinct layer in the outer edge of the mature organoid where the photoreceptors would be located.

## 6 Conclusion and future perspectives

Animal and 2D culture models have facilitated important discoveries in enlightening the pathophysiology of neurodevelopmental disorders but have limitations, particularly in recapitulating the genomic context of disease and the interregional or functional characteristics of the diseased brain. The study of human brain tissue may address some of these issues, but the limited access to human brain tissue due to ethical reasons is a significant barrier for translational neuroscientists. Human cerebral organoids and assembloids are filling this need by enabling scalable access to neural preparations (Levy and Paşca, 2023). However, owing to its unique characteristics, cerebral organoids pose significant challenges for imaging. The tight arrangement of cellular layers, millimeter-scale size, and opacity limits the study of deeper regions of the organoid by single-photon excitation-based imaging techniques such as bright-field, fluorescence, confocal, and light-sheet microscopy. We believe multiphoton microscopy (MPM) has significant advantages over other methods since it depends on non-linear excitation of cells via fluorescent and non-fluorescent mechanisms at extremely short pulse widths and provides deeper imaging using long excitation wavelengths. More importantly, we can expect multimodal MPM imaging with cerebral organoids to provide information on network dynamics deficits via calcium and voltage indicators, cell-specific migration and cell-cycle deficits, and metabolic state deficits via fluorescent and label-free MPM.

Although significant progress has recently been made in the development of optical parametric amplifiers (OPAs) that can produce longer excitation wavelengths with moderate repetition rates at short pulse widths,. current commercial ultrafast lasers have several drawbacks including high costs and sophisticated and bulky designs. As a result, democratizing these imaging techniques in neuroscience labs is extremely difficult. Alternatively, we believe that fiber lasers can be utilized instead of commercial bulky lasers since they are very small and compact, can be operated with air cooling, have a high pump-to-signal conversion efficiency, have a lower overall cost than conventional ultrafast lasers, and can provide unconventional laser characteristics such as longer wavelengths, high repetition rates, variable pulse energy, and narrow pulse widths (Srinivasan and Yildirim, 2023).

Since organoids are highly scattering compared to mouse brain which is a very common model system for different neuroscience questions, imaging of whole organoids is challenging, and it depends on their size. The size of the organoids is determined by their differentiation time. In our latest study, we examined 5-week-old organoids that ranged in size from 1 to 3 mm. We used label-free three-photon imaging and successfully imaged the organoid up to ~2 mm depth, which is the working distance of the water immersion objective lens. We also measured the effective attenuation lengths of the control and mutant organoids and discovered that their effective attenuation lengths were around two times shorter than the visual cortex of a mouse brain (Yildirim et al., 2022). Thus, we expect that the organoid size will affect the whole organoid imaging studies up to 5 weeks of differentiation where imaging of whole organoids is still feasible. After 5 weeks of differentiation, it would be difficult to image the whole organoids even with multiphoton imaging techniques. Furthermore, we predict that myelination would impact the maximal imaging depth of cerebral organoids since myelin scatters the light roughly three times more than cortical layers in the mouse brain at 1300 nm while performing three-photon microscopy (Yildirim et al., 2019, 2020).

Femtosecond pulses at longer excitation wavelengths for multiphoton microscopy also poses a challenge related to bulk heating and nonlinear photodamage. Thus, it is critical to distinguish between bulk heating, which results in one-photon interaction with the excitation wavelength and the organoids, and photodamage, which arises in nonlinear interaction between light and organoids in the focal plane. Related to bulk heating, using <250 mW at 80 MHz with 900 nm wavelength for 2PE imaging in the mouse brain was found to be safe and had no negative impact on neuronal function (Podgorski and Ranganathan, 2016). Furthermore, it was demonstrated that using 100 mW of power at 1,300 nm would be safe for performing neuronal recordings in the mouse brain with GCaMP6s (Wang and Xu, 2020) with no harmful effects on 3PE imaging. Blood perfusion can serve as a natural conductive heat dissipation technique for bulk heating, allowing the brain to sustain high average power levels. However, because cerebral organoids lack a blood perfusion system, this natural heat dissipation mechanism will be ineffective. Alternatively, convective cooling strategies such as laminar flow of water (Roy and Ben-Yakar, 2024) under a water immersion objective can be used with cerebral organoid imaging to reduce overall heating. Therefore, we expect the damage thresholds for bulk heating with 2PE and 3PE in organoids to be lower than the thresholds for mouse brain. Regarding photodamage at the focal plane, we conducted a thorough investigation that demonstrated that employing less than 2 nJ pulse energy was safe for doing neuronal recordings in the awake mouse brain at 1,300 nm wavelength, 800 kHz repetition rate, and 40 fs pulse width for 3PE microscopy (Yildirim et al., 2019). We also demonstrated that pulse energy ranging from 2 to 5 nJ affected evoked neural responses, while pulse energies greater than 5 nJ saturated GCaMP6s fluorophore. Finally, we demonstrated that pulse energy greater than 10 nJ would cause persistent damage in the mouse brain via optical breakdown. We demonstrated the safety of using <1 nJ pulse energy in 2PE microscopy for neuronal physiological recordings. For organoid imaging, the range of physiologically safe recordings for 2PE and 3PE in terms of bulk heating and photodamage would be same.

Finally, it is imperative to initiate their generation within controlled and sterile environments to ensure the sustained sterility essential for long-term multiphoton imaging of cerebral organoids. Utilizing custom-made mini-bioreactors and microfluidics not only facilitates the creation of organoids in pristine conditions but also establishes a self-contained system that minimizes the risk of external contamination. This eliminates the necessity of transferring cerebral organoids back and forth between multiwell chips and microscopes for imaging, thereby reducing potential exposure to contaminants. The implementation of such advanced technologies not only enhances sterility but also streamlines the imaging process, ensuring optimal conditions for the prolonged observation of cerebral organoid development and function. Second, multiphoton microscopy allows for deeper tissue imaging and the investigation of developmental processes that were previously difficult to examine using single-photon techniques. Third, label-free multiphoton imaging can overcome difficulties with chemical and genetically encoded fluorescent probes. Finally, we anticipate that multi-omics approaches such as spatial transcriptomics will be combined with MPM for diagnostic and therapeutic purposes such as gene therapy.

## Author contributions

MY: Writing – review & editing, Writing – original draft, Supervision, Resources, Project administration, Funding acquisition, Conceptualization. ML: Writing – review & editing, Writing – original draft, Validation, Conceptualization.
